# Discovery of an independent poor-prognosis subtype associated with tertiary lymphoid structures in breast cancer

**DOI:** 10.3389/fimmu.2024.1364506

**Published:** 2024-03-20

**Authors:** Ruiqi Liu, Xiaoqian Huang, Shiwei Yang, Wenbo Du, Xiaozhou Chen, Huamei Li

**Affiliations:** ^1^ School of Mathematics and Computer Science, Yunnan Minzu University, Kunming, China; ^2^ Department of Rheumatology and Immunology, Nanjing Drum Tower Hospital, the Affiliated Hospital of Nanjing University Medical School, Nanjing, China

**Keywords:** tertiary lymphoid structures, tumor microenvironment, breast cancer, subtypes, prognosis

## Abstract

**Introduction:**

Tertiary lymphoid structures (TLSs) are ectopic lymphoid formations that arise in non-lymphoid tissues due to chronic inflammation. The pivotal function of TLSs in regulating tumor invasion and metastasis has been established across several cancers, such as lung cancer, liver cancer, and melanoma, with a positive correlation between increased TLS presence and improved prognosis. Nevertheless, the current research about the clinical significance of TLSs in breast cancer remains limited.

**Methods:**

In our investigation, we discovered TLS-critical genes that may impact the prognosis of breast cancer patients, and categorized breast cancer into three distinct subtypes based on critical gene expression profiles, each exhibiting substantial differences in prognosis (p = 0.0046, log-rank test), with Cluster 1 having the best prognosis, followed by Cluster 2, and Cluster 3 having the worst prognosis. We explored the impact of the heterogeneity of these subtypes on patient prognosis, the differences in the molecular mechanism, and their responses to drug therapy and immunotherapy. In addition, we designed a machine learning-based classification model, unveiling highly consistent prognostic distinctions in several externally independent cohorts.

**Results:**

A notable marker gene CXCL13 was identified in Cluster 3, potentially pivotal in enhancing patient prognosis. At the single-cell resolution, we delved into the adverse prognosis of Cluster 3, observing an enhanced interaction between fibroblasts, myeloid cells, and basal cells, influencing patient prognosis. Furthermore, we identified several significantly upregulated genes (CD46, JAG1, IL6, and IL6R) that may positively correlate with cancer cells' survival and invasive capabilities in this subtype.

**Discussion:**

Our study is a robust foundation for precision medicine and personalized therapy, presenting a novel perspective for the contemporary classification of breast cancer.

## 1 Introduction

As a widely utilized high-throughput sequencing method, RNA sequencing technology empowers scientists to analyze gene expression at the whole transcriptome level comprehensively. In recent years, the rapid advancements in next-generation sequencing technologies have yielded extensive biomedical data on cancer, including information on cancer genomes, transcriptomes, proteomes, and the tumor immune microenvironment. Leveraging cutting-edge analytical techniques in machine learning and deep learning, it has become possible to delve deeply into data and unearth insights into the pathogenesis, intrinsic heterogeneity, effective therapeutic targets, and other potential aspects of specific cancers within large-scale research cohorts (1–3). This process aids in achieving early and precise cancer diagnoses, selecting personalized medical treatment strategies, and accurately predicting therapeutic responses and prognostic risks.

According to the latest cancer statistics in the United States published in CA Cancer J Clin, breast cancer (BC) is the first malignant tumor with the highest incidence rate in women currently, and by 2023, it will have the highest number of new cases of all cancer types. Among all female cancer patients, the incidence rate of breast cancer is as high as 31%, and the mortality rate has been ranked second, which seriously jeopardizes women’s lives and health (4, 5). Based on gene expression profile characteristics, breast cancer can be classified into five intrinsic molecular subtypes, which are known as PAM50 typing. However, gene sequencing is difficult to promote in the clinic due to its high cost, time-consuming, and other drawbacks. The widely used typing method is based on immunohistochemistry (IHC), which detects the expression of Estrogen Receptor (ER), Progesterone Receptor (PR), HER2, and Ki-67. IHC typing has a large margin of error and low robustness and requires a biopsy of the patient, for which some patients are not suitable (6, 7).

In recent years, some prognostic gene signatures associated with breast cancer have been identified in several studies and applied to clinical practice (8, 9). There is a long-standing hypothesis that secondary lymphoid organs (SLOs) are the main sites of anti-tumor immune responses (10). However, due to the distance between SLOs and tumor tissues, immune cells can only migrate inside the tumors to function. Nevertheless, recent findings have identified certain anti-tumor immune response sites within tumor tissues, known as tertiary lymphoid structures (11). TLSs are ectopic lymphoid structures formed at sites of chronic inflammation in non-lymphoid tissues (12), composed of a variety of cells such as T cells, B cells, follicular dendritic cells (FDCs), and other cells with high endothelial venules (HEV). It has been shown that TLSs play a crucial role in controlling tumor invasion. For example, the correlation between high density of TLSs and more prolonged overall survival (OS) and disease-free survival (DFS) has been demonstrated in a large number of solid tumors such as lung, colorectal, liver, breast, pancreatic, and melanoma (13–20). Not only that, TLSs in tumor tissues also play a crucial role in anti-tumor immune response and are related to the prognosis of immunotherapy closely (21–23).

Given the close relationship between TLSs and cancer prognosis, we looked at the expression levels of TLS-related genes in breast cancer. We assessed their integrated correlation with tumor microenvironment (TME) heterogeneity and clinical prognosis. Specifically, we used TLS-related gene expression profiles to typify breast cancer. Further, we explored the intrinsic heterogeneity characteristics of TLS-derived breast cancer subtypes, such as analyzing the prognosis of different subtypes, enriched genes and pathways, differences in molecular mechanisms, searching for subtype-specific biomarkers, etc., which, from the perspective of TLSs, provides an insightful understanding of the pathogenesis and progression of breast cancer, and also provides the support and foundation for precision medicine and personalized treatment.

## 2 Materials and methods

### 2.1 Molecular data and clinical information on breast cancer

In this study, transcriptome expression data and clinical information were acquired for a total of 1215 breast cancer samples from The Cancer Genome Atlas Project (TCGA). Copy number variant data, calculated using the Illumina platform based on the GISTIC2 method, were downloaded from the UCSC Xena database (https://xenabrowser.net/datapages/). The METABRIC dataset, encompassing transcriptome expression data and clinical information for 1904 breast cancer patients, was downloaded from cbio-portal (https://www.cbioportal.org) (24). Additionally, external independent breast cancer datasets GSE19615 (n = 115) and GSE20685 (n = 327) (25, 26), the 10X single-cell RNA sequencing dataset GSE195665 (n = 167) (27), along with the cohort of triple-negative breast cancer patients receiving anti-angiogenic immunotherapy GSE103668 (n = 21) (28), were downloaded from Gene Expression Omnibus (GEO).

### 2.2 Consensus clustering for investigating potential breast cancer subtypes

We implemented unsupervised consensus clustering with the parameter “clusterAlg = pam, distance = euclidean, pItem = 0.8” using the “ConsensusClusterPlus” R software package (version 1.64.0) (29). This approach enabled the adjustment of the cluster number from 2 to 6, facilitating the identification of the most stable consensus matrix and the most distinct cluster assignment during the iterative clustering process.

### 2.3 Computational index for breast cancer-related events and immune microenvironment

We used the “method = ssgsea” function from the “GSVA” software package (version 1.48.0) to ascertain the activity scores of TLS-related genes in patients (30). To evaluate any significant variations in overall survival (OS) among subtypes, we conducted Kaplan-Meier (KM) survival analysis and applied the log-rank test using the “survival” software package (version 3.5.5) (31). To identify differentially expressed genes (DEGs) within the TLS-derived subtypes, we employed the FindAllMarkers function integrated within the “Seurat” software package (version 4.3.0) (32). Genes exhibiting adjusted *p* < 0.05 and a log2FC > 1 were classified as significantly differentially expressed. For an in-depth exploration of the distinct biological processes and pathways manifesting significant differential expression among the subtypes, we harnessed the bitr function of the “clusterProfiler” software package (version 4.0.5) to convert gene symbols to Entrez IDs (33). Subsequently, we conducted an enrichment analysis using the enrichGO function with the “ont = BP” parameter, as well as the enrichKEGG function, to elucidate and analyze the enriched Gene Ontology (GO) and Kyoto Encyclopedia of Genes and Genomes (KEGG) pathways. GO and KEGG terms demonstrating adjusted *p* < 0.05 were deemed significantly enriched. We leveraged the “ESTIMATE” software package (version 1.0.13) to estimate stromal scores, immune scores, and tumor purity in patients (34), the immune infiltration tool “CIBERSORT” was used for assessing the relative abundance of 22 immune cell types in patients (35). Furthermore, we used the “genefu” software package (version 2.32.0) to predict the PAM50 type of patients in the single-cell dataset (GSE195665) (36).

### 2.4 Building machine learning models to predict Cluster 1&2 and Cluster 3 subtypes of breast cancer patients

Considering that Cluster 3 identified in the TCGA breast cancer cohort has the worst prognosis, we introduced the XGBoost model to differentiate between Cluster 1&2 and Cluster 3 categories of individual breast cancer patients. The TCGA breast cancer cohort was randomly divided into training and testing sets according to 7:3, and the TLS-critical genes obtained from Lasso regression were used as features in the training set. The XGBoost model was applied to the training set using the “xgboost” function in the “XGBoost” R package (version 1.7.5.1), with parameters set to “nfold = 10, objective = binary: logistic, max. depth = 8, eval_metric = logloss”, the model with the highest AUC value was kept. The model performance was tested using the testing set and other external independent datasets (METABRIC dataset, GSE195665, GSE20685, GSE195665, and GSE103668).

### 2.5 Performing cell communication analysis using CellChat

In the single-cell dataset, we used the CellChat package (version 1.6.1) to analyze intercellular communication in Cluster 1&2 and Cluster 3 (37). CellChat is a specialized tool for analyzing intercellular communication by integrating known intercellular communication databases and single-cell RNA sequencing data, assisting in revealing potential interactions between cell populations and contributing to the understanding of the dynamic characteristics of intercellular communication in biological processes.

### 2.6 Statistical analysis

All statistical analyses were performed in R4.3.0. Standard statistical tests such as Student’s t-test, Wilcoxon rank sum test, log-rank test, and Cox proportional risk regression were used to analyze the expression and clinical data, and *p* < 0.05 was considered statistically significant.

## 3 Results

### 3.1 Heterogeneity of TLSs in breast cancer and potential prognostic value

**Figure 1:**
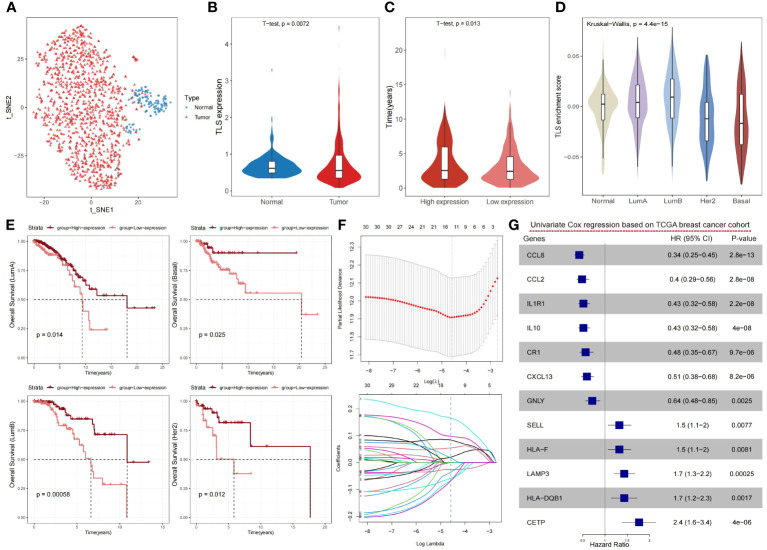
Expression levels of TLS-related genes reveal the heterogeneity of the tumor microenvironment and its impact on prognosis in breast cancer. **(A)** The t-SNE plot showing a projection of breast cancer samples based on TLS-related genes, with each point representing a sample; tumor samples are shown in red and normal samples in blue. **(B)** Violin plot showing the differences in the expression levels of TLS-related genes in normal and cancer samples of the breast cancer cohort. P-value was obtained by t-test. **(C)** Violin plot showing the distribution of overall survival time between high- and low-expression groups of TLS-related genes in the breast cancer cohort. The high- and low-expression groups were divided by the median, and the P value was obtained by t-test. **(D)** Violin plot showing the distribution of enrichment scores of TLS-related genes in PAM50 typing, with P-values obtained by Wilcoxon rank sum test. **(E)** Kaplan-Meier survival curves between the high- and low-expression groups of TLS-related genes in PAM50 typing. P-values were calculated by log-rank test. **(F)** The LASSO algorithm was used to process the TLS-related genes, and 12 critical genes were screened for subsequent analysis. **(G)** Forest plot showing the prognostic impact of the 12 TLS-critical genes on the TCGA breast cancer cohort as determined by univariate Cox regression analysis.

TLSs are an integral part of anti-tumor immunity, and its density is closely related to patient survival, prognosis, and recurrence. Based on the existing studies on TLSs, we included 79 TLS-related genes by reviewing the literature (11, 18, 19, 21, 38–42) to analyze 1,215 breast cancer samples from TCGA (**Supplementary Table S1**). These genes are mainly chemokine- and immune cell-associated genes which are closely related to the formation of tertiary lymphoid structures. In the TCGA breast cancer cohort, we observed a noteworthy disparity in the distribution of TLSs between tumor and normal samples (**Figure 1A**), and there were notable distinctions in the expression levels of TLS-related genes between tumor and normal samples (**Figure 1B**). The high-expression group had a longer overall survival time than the low-expression group (**Figure 1C**). To validate the strong relationship between TLSs and the prognosis of breast cancer patients, we compared the relationship between the TLS-related gene activity score and PAM50, the classical staging of breast cancer. The results showed that the Her2 and Basal subtypes, which had a poorer prognosis, had lower TLSs (**Figure 1D**). Although PAM50 typing can be used to predict the prognosis of patients, such as LumA has a better prognosis, whereas Her2 and Basal have a worse prognosis, we were still able to differentiate between better and worse prognostic groups from each of the PAM50 subtypes, based on the level of TLS-related gene expression. Similarly, the prognosis of the high-expression group was significantly better than the low-expression group, demonstrating the good prognostic predictive ability of TLS-related genes (**Figure 1E**).

To obtain more effective and reliable gene signatures for predicting the prognosis, we screened the above 79 TLS-related genes using LASSO regression and obtained 12 TLS-critical genes (**Figure 1F**). The univariate Cox regression analysis showed that all critical genes significantly correlated with prognosis. CCL8, CCL2, IL1R1, IL10, CR1, CETP, and GNLY were potential protective factors, whereas SELL, HLA-F, LAMP3, HLA-DQB1, CXCL13 were potential risk factors (**Figure 1G**). In summary, TLSs show significant heterogeneity in breast cancer and can impact the prognosis of patients.

### 3.2 Determination of breast cancer subtypes based on TLS-critical genes

**Figure 2:**
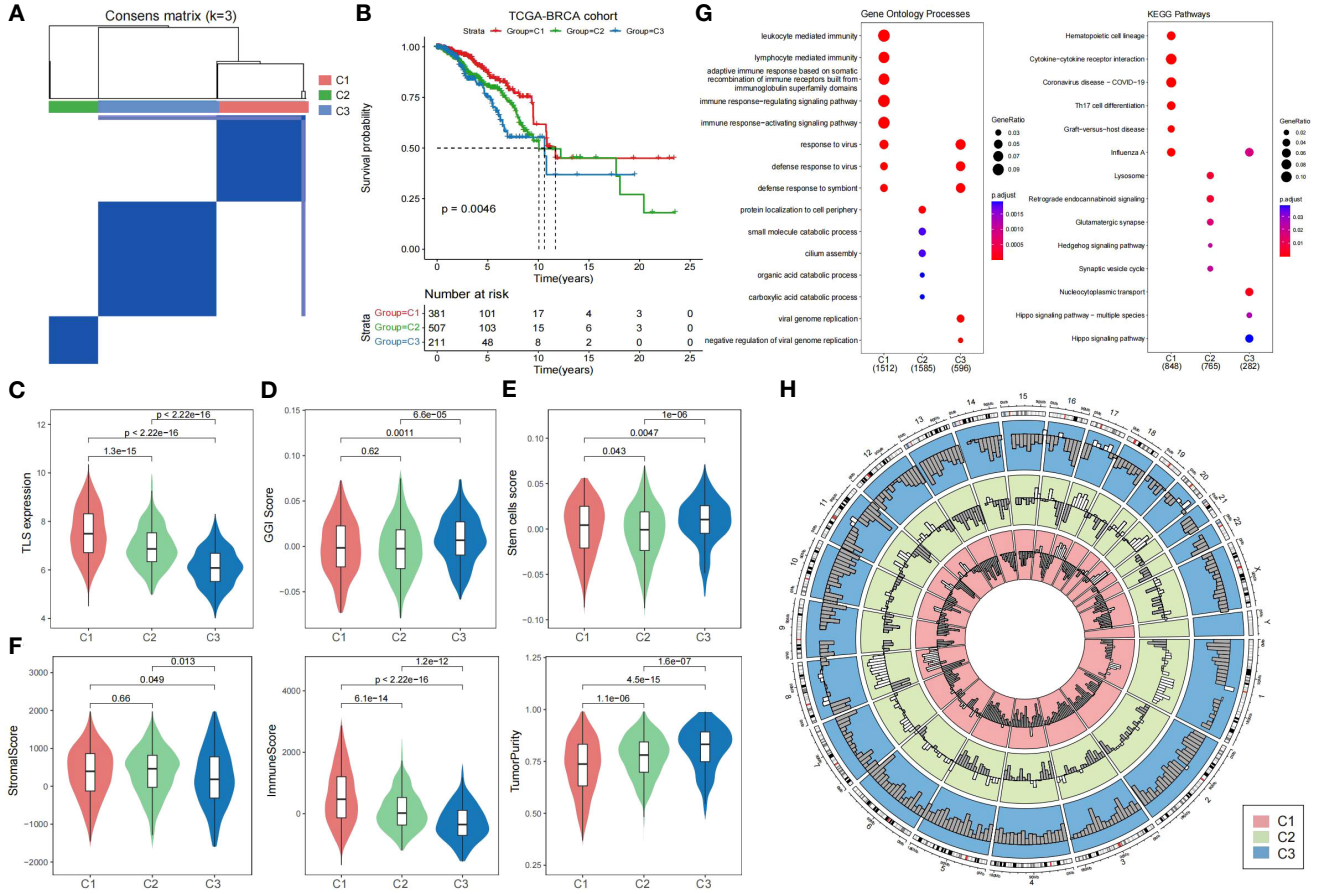
Heterogeneity of TLS-derived breast cancer subtypes. **(A)** Heatmap showing the consensus clustering matrix of the TCGA breast cancer cohort based on the TLS-critical gene expression matrix. **(B)** KM curves showing significant differences in prognosis among the three TLS-derived subtypes. P-value was obtained by log-rank test. **(C)** Violin plot showing differences in the expression of TLS-critical genes among TLS-derived subtypes. P-values were obtained by t-test. **(D)** Violin plot showing the GGI of the TLS-derived subtypes, with P-value derived by t-test. **(E)** Violin plot showing the stem cell signature gene activity scores of the TLS-derived subtypes. The P-value was derived by t-test. **(F)** Violin plot showing the distribution of different biological characteristics in the TLS-derived subtypes, including immune score, stromal score, and tumor purity, with P-values derived by t-test. **(G)** Bubble plot showing enriched gene ontology terms and KEGG pathways. **(H)** Circle plot showing the copy number variation of the TLS-derived subtypes, with white indicating copy number increase and gray indicating copy number deletion.

To investigate the prognostic value of TLSs in breast cancer more precisely, we performed a consensus clustering analysis on TLS-critical gene expression profiles in the TCGA breast cancer cohort. We categorized breast cancer patients into three subtypes: Cluster 1, Cluster 2, and Cluster 3 (**Figure 2A**; **Supplementary Table S2**; see Materials and Methods). The results showed that TLS-derived breast cancer subtypes reflected significant variability in prognosis, with Cluster 1 having the best prognosis, followed by Cluster 2, and Cluster 3 having the worst prognosis (**Figure 2B**). By comparing the expression levels of TLS-critical genes in the three subtypes, it can be found that TLS-critical genes were expressed at the highest level in Cluster 1, followed by Cluster 2, and at the lowest level in Cluster 3 (**Figure 2C**), which is in line with the results of the previous analyses: higher TLSs reflects better prognosis. To further validate the characteristics of the three subtypes, we introduced the genomic grade index (GGI), which is a gene expression signature designed to enhance the histologic grade assessment (43). It was found that Cluster 3 had the highest GGI (**Figure 2D**), implying that Cluster 3 had a worse prognosis and reduced responsiveness to immunotherapy treatment. We also calculated the stem cell characteristic gene activity scores of the three subtypes. Stem cell characteristic gene activity score is strongly associated with tumor aggressiveness, risk of recurrence and patient prognosis. Cluster 3 had the highest score (**Figure 2E**), and the resulting subtype heterogeneity may provide the basis for selecting treatment-resistant clones, leading to poor clinical outcomes. Not only that, we further analyzed the stromal score, tumor purity and immune score of these three subtypes to explore the tumor microenvironment characteristics of the three subtypes. We found that Cluster 3 had the lowest immune scores, the stromal scores, and the highest tumor purity (**Figure 2F**), implying that among the TLS-derived subtypes, Cluster 3 had the lowest immune cell infiltration and activity, the highest tumor cell density, and tumor invasiveness.

To elucidate the biological functions and pathways involved in TLS-derived subtypes, we performed differential gene expression analysis, as well as GO and KEGG functional enrichment analysis (**Figure 2G**). Cluster 1 was significantly associated with the immune system and organisms’ response to external pathogens or other organisms. Cluster 2 was significantly associated with protein localization to the cell periphery, small molecule degradation, cilia assembly, and organic acid catabolism, which play essential roles in maintaining normal cellular function. Cluster 3 was significantly associated with viruses replicating their genomes and the negative regulatory processes that regulate viral genome replication, involving key processes in viral biology (**Supplementary Table S3**). Further functional enrichment analysis showed that Cluster 1 was significantly associated with hematopoietic cell lineage, cytokine-cytokine receptor interactions, and Th17 cell differentiation pathways, Cluster 2 was significantly associated with lysosomal, Hedgehog signaling and synaptic vesicle cycling pathways, and Cluster 3 was associated with nucleoplasmic transfer pathway and Hippo signaling pathway (**Supplementary Table S4**). Notably, Hippo is a very conserved signaling pathway responsible for regulating cell proliferation and apoptosis, and its major components are mutated and dysregulated in a variety of cancers, promoting the development of malignant tumors. It has been demonstrated that transcriptional coactivators downstream of the Hippo signaling pathway, including YAP/TAZ, promote the proliferation of breast cancer cells by stabilizing the KLF5 transcription factor, survival, and tumor growth, leading to poor prognosis of breast cancer (44).

In addition, we obtained copy number variation data for the TCGA breast cancer cohort at the GDC Xena Hub (https://xenabrowser.net/datapages/). By looking at the copy number variation of TLS-derived subtypes, we found that the genetic variant profiles also exhibited significant heterogeneity. More copy number amplifications occurred in Cluster 1&2, while more copy number deletions occurred in Cluster 3 (**Figure 2H**). Specifically, on chromosomes 1, 8, 11, 16, 17, and 20, Cluster 1&2 showed more copy number amplification, while Cluster 3 showed only a few copy number amplifications. Besides, on chromosomes 3, 4, 5, 6, 7, 10, 12, 19, and X, copy number amplification was observed in Cluster 1&2, whereas in Cluster 3, there was no copy number amplification but only copy number deletion. This difference also reflects the molecular heterogeneity within breast cancer, and these molecular changes may lead to different characteristics and functions of tumor cells, which affects the tumor’s treatment response and prognosis, leading to differences in the response of different subtypes to treatment. Therefore, this difference could be considered when developing treatment regimens. Gene amplification is usually associated with the overproliferative capacity of tumor cells, and for Cluster 1&2, which has more gene amplification, inhibiting the overproliferative capacity of tumor cells may be an effective therapeutic strategy. On the contrary, gene deletions may lead to reduced tumor suppressor function, and for Cluster 3 with more gene deletions, focusing on restoring tumor suppressor function may be another effective therapeutic strategy.

### 3.3 Prognostic value of TLS-derived breast cancer subtypes

**Figure 3:**
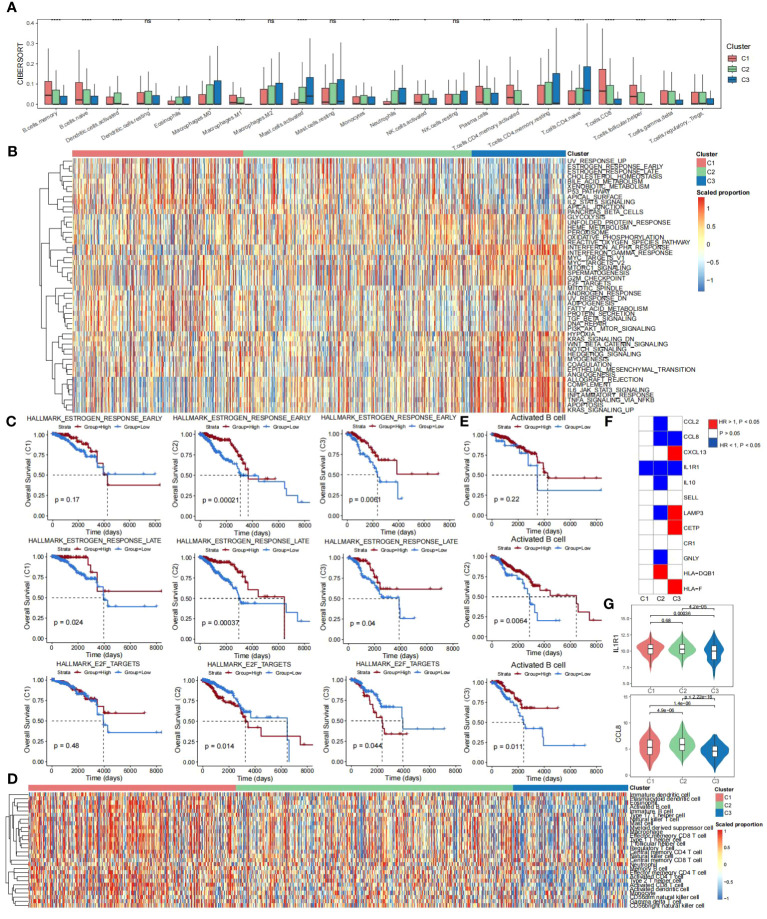
Prognostic differences in TLS-derived breast cancer subtypes. **(A)** Box line plot showing the differences in immune infiltration among the TLS-derived subtypes, P-values were derived by Wilcoxon rank sum test. ns, not significant; *p < 0.05; **p < 0.01; ***p < 0.001; ****p < 0.0001. **(B)** Heatmap showing activity scores of cancer signature pathways for the TLS-derived subtypes. These pathways were obtained from the MSigDB database. **(C)** KM curves showing the effect of the activity of cancer signature pathways on the prognosis of TLS-derived subtypes. P-values were obtained by log-rank test. **(D)** Heatmap showing the effect of the activity of 28 immune cell genomes on the prognosis of TLS-derived subtypes. **(E)** KM curves showing the effect of immune cell activity on the prognosis of patients with TLS-derived subtypes. P-values were obtained by log-rank test. **(F)** Heatmap showing the effect of the expression of TLS-critical genes on the prognosis of TLS-derived subtypes. P-values were obtained by log-rank test. **(G)** Violin plot showing the differences in the distribution of IL1R1 and CCL8 expression levels in TLS-derived subtypes. P-values were obtained by t-test.

To investigate the differences in immune infiltration of TLS-derived breast cancer subtypes, we used the CIBERSORT cell deconvolution tool to assess the relative abundance of 22 immune cell types in three subtypes. The results showed that Cluster 3 had the lowest degree of immune infiltration (**Figure 3A**). Next, we investigated the impact of potential interactions between cancer signature pathways and TLS-derived subtypes on patients and found that some pathways such as HALLMARK_UV_RESPONSE_UP, HALLMARK_ESTROGEN_RESPONSE_EARLY, and other signature signaling pathways had lower activity levels in Cluster 3 (**Supplementary Table S5**), may be positively correlated with survival, while HALLMARK_KRAS_SIGNALING_UP, HALLMARK_APOPTOSIS, HALLMARK_TNFA_SIGNALING_VIA_NFKB, and other signature signaling pathways were more active in Cluster 3, may be negatively correlated with survival (**Figure 3B**). To verify this speculation, we selected three signature signaling pathways, HALLMARK_ESTROGEN_RESPONSE_EARLY, HALLMARK_ESTROGEN_RESPONSE_LATE, and HALLMARK_E2F_TARGETS, and observed the KM curves of their OS. The results were consistent with our speculation that the high activity of HALLMARK_ESTROGEN_RESPONSE_EARLY versus HALLMARK_ESTROGEN_RESPONSE_LATE in Cluster 3 represented a better prognosis, and HALLMARK_E2F_TARGETS in Cluster 3 High activity, on the other hand, was associated with a worse prognosis (**Figure 3C**). We further evaluated the activity of 28 immune cells (45) in TLS-derived subtypes, and it could be seen that Cluster 1 and Cluster 2 were immunized to a better extent than Cluster 3 (**Figure 3D**; **Supplementary Table S6**). We chose Activated B cells with low activity in Cluster 3, observed the KM curve of their OS, and found that the lower activity of Activated B cells led to a poor prognosis of Cluster 3 (**Figure 3E**). All of these results suggest that TLS-derived breast cancer typing heterogeneity can influence breast cancer prognosis and can be utilized as a feasible factor to predict the prognosis. In addition, we investigated the association between TLS-critical genes and the survival of TLS-derived subtypes and found IL1R1 and CCL8 are protective factors (HR < 1, *p* < 0.05) (**Figure 3F**). IL1R1 and CCL8 are implicated in the regulation of inflammation and immune response. We next compared their expression differences in the three subtypes and found the lowest expression level of IL1R1 and CCL8 in Cluster 3, which may have contributed to the poor prognosis (**Figure 3G**).

### 3.4 Prediction of TLS-derived subtypes in external datasets using XGBoost

**Figure 4:**
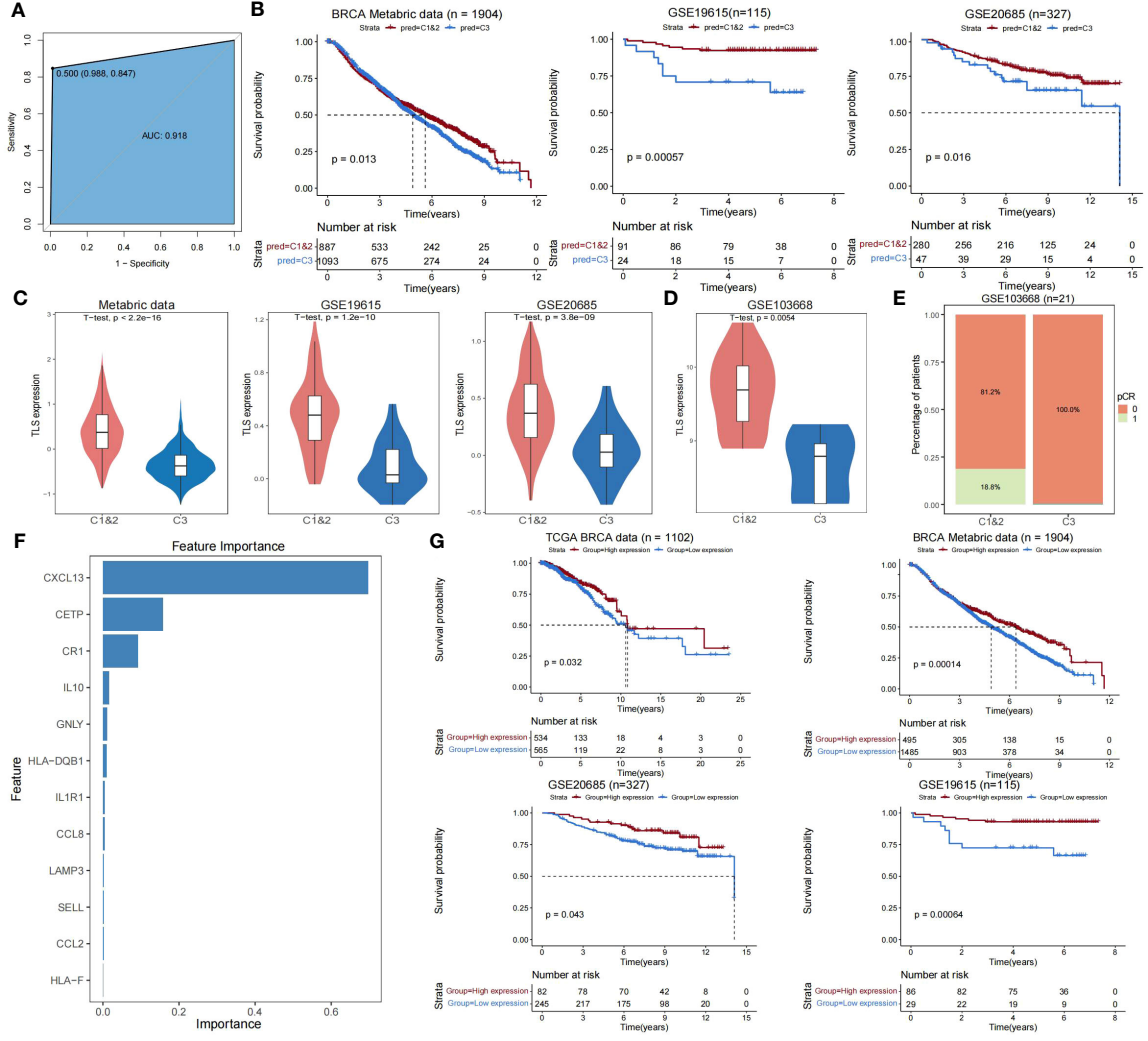
Validating the reliability of TLS-derived subtypes in external datasets. **(A)** ROC curve showing the accuracy of the XGBoost classifier in predicting specific samples in the training set. **(B)** Kaplan-Meier survival curves for Cluster 1&2 versus Cluster 3 overall survival predicted using XGBoost. P-values were obtained by log-rank test. **(C)** Violin plot showing the difference in expression levels of TLS-critical genes in Cluster 1&2 versus Cluster 3 predicted using XGBoost. P-values were obtained from the t-test. **(D)** Violin plot showing the difference in expression levels of TLS-critical genes in Cluster 1&2 versus Cluster 3 using XGBoost predictive immunotherapy cohorts. P-values were obtained by t-test. **(E)** Stacked bar graph showing the comparison of disease treatment effects between Cluster 1&2 versus Cluster 3 in the predicted immunotherapy cohort using XGBoost. pCR = 0 indicates this sample did not achieve pCR and pCR = 1 indicates this sample achieved pCR. **(F)** Bar graph showing the importance ranking of 12 TLS-critical genes (sorted by gain index) in the training set. **(G)** KM curves showing the effect of CXCL13 on the prognosis of breast cancer patients. The P-value was obtained by log-rank test.

To develop an accurate and unsupervised clustering-independent method for predicting TLS-derived subtypes in breast cancer patients, we trained the XGBoost model for predicting Cluster 1&2 and Cluster 3 subtypes based on the breast cancer cohort of TCGA and the 12 TLS-critical genes obtained by using Lasso regression previously (see Materials and Methods). Our model achieved an accuracy of 0.92 in predicting the training set of TCGA (**Figure 4A**). Applying our model to external datasets of breast cancer (Metabric dataset, GSE19615, GSE20685) and combining it with clinical information, we were able to demonstrate that the predicted Cluster 1&2 and Cluster 3 subtypes differed significantly in prognosis (**Figure 4B**). The expression pattern of TLS-critical genes is consistent with the TCGA-BRCA cohort (**Figure 4C**). Furthermore, due to the extensive attention that TLSs have received in immunotherapy in recent years (21–23), we additionally collected a dataset of 21 triple-negative breast cancer patients who were treated with cisplatin and bevacizumab in a neoadjuvant setting (GSE103668), to test the predictive value of TLS-derived subtypes. Our model divided the cohort into two groups, Cluster 1&2 and Cluster 3, the differences in the expression of TLS-critical genes were also consistent with the other datasets tested (**Figure 4D**). Notably, while 18.8% of patients in Cluster 1&2 achieved pathologic complete remission (pCR), no patients in Cluster 3 achieved pCR, indicating a worse response to the neoadjuvant setting in Cluster 3 (**Figure 4E**).

We next analyzed the importance of TLS-critical genes in the XGBoost prediction model and found that CXCL13 had the highest importance (**Figure 4F**; **Supplementary Table S7**). Combining clinical information, we found that CXCL13 effectively predicted patient prognosis in the TCGA-BRCA cohort and external datasets (Metabric dataset, GSE19615, GSE20685) (**Figure 4G**). Previous studies have identified that TLS production requires continuous chronic stimulation, its maintenance depends on some molecules, including CXCL13 (43, 46), and increasing CXCL13 levels promotes the formation of TLSs (47–49). These results suggest that CXCL13 is an essential biomarker for clinical studies of Cluster 3, which may be necessary for improving breast cancer patients’ prognosis.

### 3.5 Single-cell perspective of TLS-derived subtypes

**Figure 5:**
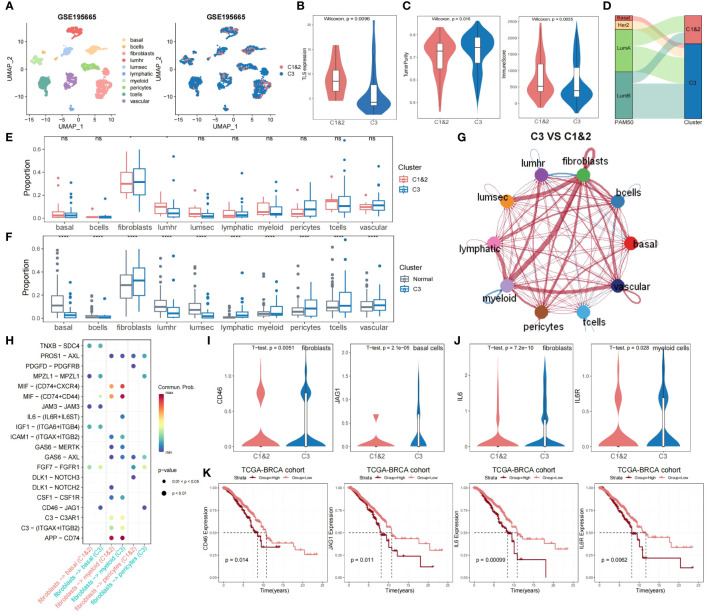
Single-cell profiling of the immune microenvironment and TLS-derived subtypes interactions. **(A)** The predictive result of the breast cancer single-cell dataset (n = 44). **(B)** Violin plot showing the difference in expression levels of TLS-critical genes in Cluster 1&2 versus Cluster 3 predicted using XGBoost. The P-value was obtained by Wilcoxon rank sum test. **(C)** Violin plot showing the difference in tumor purity and immune scores of Cluster 1&2 versus Cluster 3 predicted using XGBoost. P-values were obtained by Wilcoxon rank sum test. **(D)** Sankey plot showing the association of Cluster 1&2 versus Cluster 3 with PAM50 typing. **(E)** Grouped boxplot showing the cellular proportion of Cluster 1&2 and Cluster 3 samples. P-values were obtained from t-tests. ns, not significant, *p < 0.05. **(F)** Grouped boxplot showing the cellular proportion of normal and Cluster 3 samples. P-values were obtained from t-tests. ****p < 0.0001. **(G)** Interaction map depicting the ligand-receptor interactions within the immune microenvironment. The width of edges represents the relative interaction strength. Red colored edges represent increased signaling in Cluster 3 compared to Cluster 1&2, while blue colored edges represent decreased signaling in Cluster 3 compared to Cluster 1&2. **(H)** Bubble plot showing the significant interactions between receptor and ligand genes among fibroblasts, basal cells, myeloid cells, and pericytes in Cluster 1&2 and Cluster 3. **(I)** Violin plot showing the differences in the distribution of CD46 expression levels in fibroblasts and JAG1 expression levels in basal cells of both Cluster 1&2 and Cluster 3. P-values were obtained by t-test. **(J)** Violin plot showing the differences in the distribution of IL6 expression levels in fibroblasts and IL6R expression levels in myeloid cells of both Cluster 1&2 and Cluster 3. P-values were obtained by t-test. **(K)** KM curves showing the effect of CD46, JAG1, IL6, and IL6R on the prognosis of TCGA-BRCA cohort. P-values were obtained by log-rank test.

To further validate the significance of TLS-derived subtypes, we applied the model to the single-cell dataset (GSE195665) from the GEO database. We performed a detailed analysis of the single-cell transcriptome data of 834,356 cells from 167 samples, including steps of downsampling, quality control, batch-effects correction, and normalization, and finally, we used XGBOOST to predict TLS typing for 44 tumor samples (**Figure 5A**). By comparing Cluster1&2 and Cluster3, we observed that the differences in expression patterns of TLS-critical genes, immune scores, and tumor purity were all consistent with our results obtained in other datasets (**Figures 5B, C**). Utilizing the genefu package to predict PAM50 typing for tumor samples from the single-cell dataset, we noted that samples predicted as Basal and Her2 were also predicted as Cluster 3 (**Figure 5D**). These findings re-emphasize the poor prognosis of Cluster 3 and confirm the feasibility of our typing strategy from a single-cell perspective.

We compared cellular fraction differences between Cluster 1&2 and Cluster 3, as well as between normal samples and Cluster 3. Notably, Cluster 3 exhibited elevated fibroblasts abundance and diminished lumhr cells abundance (**Figures 5E, F**). Cellchat analysis demonstrated that, compared to Cluster 1&2, fibroblasts in Cluster 3 had significantly stronger interactions with other cells, especially with myeloid cells, but weaker interactions with lumhr cells (**Figure 5G**; see Materials and Methods). Further analysis of receptor and ligand genes showed that the interaction of CD46-JAG1 and IL6-(IL6R+IL6ST) was significantly enhanced in Cluster 3 (**Figure 5H**). We observed the expression patterns of CD46 and JAG1 in fibroblasts, pericytes, and basal cells, as well as IL6, IL6R, and IL6ST in fibroblasts and myeloid cells. Within Cluster 3, the expression of CD46 was significantly increased in fibroblasts, JAG1 in basal cells, IL6 in fibroblasts, and IL6R in myeloid cells (**Figures 5I, J**). The KM survival curves showed that CD46, JAG1, IL6, and IL6R were all risk factors that lead to poor prognosis in breast cancer patients (**Figure 5K**), which implies that the enhanced interaction between CD46-JAG1 leads to poor prognosis in breast cancer patients, along with the IL6-(IL6R+IL6ST) axis. Therefore, we hypothesized that CD46 and IL6 were upregulated in fibroblasts, with enhanced interactions with basal cells and myeloid cells, leading to upregulation of JAG1 and IL6R expression, ultimately contributing to the poor prognosis of Cluster 3.

## 4 Discussion

TLSs are increasingly recognized as a focus of anti-tumor immunity, and it has been demonstrated that a higher number of mature TLSs in tumors is associated with favorable outcomes in various cancers. We explored the breast cancer microenvironment using TLS-related genes and found the heterogeneity of TLSs in breast cancer, which supports our TLS-derived typing of breast cancer. Therefore, we used consensus clustering analysis on the TLS-critical gene expression profiles of the TCGA breast cancer cohort and obtained three subtypes (Cluster 1, Cluster 2, and Cluster 3), and found that the three subtypes differed significantly in prognoses. Gene ontology annotation and pathway analysis revealed these three subtypes were associated with different biological processes. Calculation of the activity scores of signature cancer signaling pathways and immune cell genomes in TLS-derived subtypes revealed that the heterogeneity of TLS-derived subtypes may affect prognosis and may be utilized as a viable predictor of prognosis. By further evaluating the degree of immune infiltration, stromal scores, tumor purity, GGI, and copy number variant differences of the TLS-derived subtypes, the results showed that Cluster 3 had the lowest immune scores, stromal scores, the highest tumor purity, and the highest GGI, which were all consistent with its poor prognosis. In addition, we identified many gene deletions in Cluster 3, suggesting significant heterogeneity in the characterization of genetic variation in TLS-derived subtypes at the genomic level. The above results suggest that TLS-derived subtypes could provide a new strategy for current breast cancer typing.

To validate the reliability of TLS-derived subtypes, we developed a prediction model using XGBoost to predict Cluster 1&2 versus Cluster 3 in other externally independent datasets. The model was evaluated to perform well in the TCGA training set (accuracy = 0.92). In other externally independent breast cancer cohorts, the subtypes predicted by our model showed prognostic differences and significant gene expression differences consistent with the expected pattern. This supports TLSs for typing studies in a broader range of breast cancer cohorts. In addition, we found that CXCL13 is the most critical marker gene in the Cluster 3 subtype. As mentioned in a previous study, the process of TLS formation is as follows: activation of local fibroblasts, recruitment of immune cells to maturation, and activated fibroblasts in the first step promote the recruitment and aggregation of lymphocytes through the secretion of the pro-angiogenic factors CXCL13 and CCL19 (50), so a better prognosis can be achieved by increasing CXCL13 levels to promote TLSs formation.

Finally, we applied the model to a breast cancer single-cell dataset. Again, the Cluster 3 subtype showed a worse prognosis, higher tumor purity, and lower immune scores. After observing the cellular components and intercellular communication, we hypothesized that in Cluster3, CD46, and IL6 were upregulated in fibroblasts, with enhanced interactions with basal cells and myeloid cells, leading to upregulation of the expression of JAG1 and IL6R, ultimately contributing to the poor prognosis of Cluster 3. Relevant research has demonstrated that the inflammatory cytokines IL6/IL6R amplify the signaling of the Notch-Jagged pathway, which stimulates the generation of hybrid epithelial-mesenchymal cancer stem cells, and that inhibition of CD46 gene expression reduces the effects of proliferation, invasion, and migration capacity of breast cancer cells. Knockdown of the JAG1 gene significantly reduced the potential for tumor organogenesis in triple-negative breast cancer (TNBC) cells, and JAG1-mediated adaptive resistance in Her2 breast cancer cells led to tumor recurrence (51). Overall, these studies highlight the critical roles of IL6 and IL6R, as well as CD46 and JAG1, in the survival and invasive capacity of cancer cells, and their broad promise as targets for antitumor therapies, as well as exploring the possibility of combining them with other therapeutic agents.

## Data availability statement

The datasets presented in this study can be found in online repositories. The names of the repository/repositories and accession number(s) can be found in the article/**Supplementary Material**.

## Author contributions

RL: Data curation, Formal analysis, Visualization, Writing – original draft, Writing – review & editing. XH: Investigation, Formal analysis, Writing – review & editing. SY: Writing – review & editing. WD: Writing – review & editing. XC: Conceptualization, Software, Supervision, Writing – review & editing. HL: Conceptualization, Funding acquisition, Methodology, Writing – review & editing.

## Supplementary material

Supplementary Table S1 List of TLS-related genes.

Supplementary Table S2 Results of consensus Clustering.

Supplementary Table S3 GO terms enriched in TLS-derived subtypes.

Supplementary Table S4 KEGG pathways enriched in TLS-derived subtypes.

Supplementary Table S5 Activity scores of cancer signature pathways of TLS-derived subtypes.

Supplementary Table S6 Activity scores of immune cell gene set of TLS-derived subtypes.

Supplementary Table S7 Importance of TLS-critical genes in the XGBoost prediction model.
